# Intrinsic Noise Improves Speech Recognition in a Computational Model of the Auditory Pathway

**DOI:** 10.3389/fnins.2022.908330

**Published:** 2022-06-08

**Authors:** Achim Schilling, Richard Gerum, Claus Metzner, Andreas Maier, Patrick Krauss

**Affiliations:** ^1^Laboratory of Sensory and Cognitive Neuroscience, Aix-Marseille University, Marseille, France; ^2^Neuroscience Lab, University Hospital Erlangen, Erlangen, Germany; ^3^Cognitive Computational Neuroscience Group, Friedrich-Alexander-University Erlangen-Nuremberg (FAU), Erlangen, Germany; ^4^Department of Physics and Center for Vision Research, York University, Toronto, ON, Canada; ^5^Friedrich-Alexander-University Erlangen-Nuremberg (FAU), Erlangen, Germany; ^6^Pattern Recognition Lab, Friedrich-Alexander-University Erlangen-Nuremberg (FAU), Erlangen, Germany; ^7^Linguistics Lab, Friedrich-Alexander-University Erlangen-Nuremberg (FAU), Erlangen, Germany

**Keywords:** speech processing, auditory perception, hearing loss, stochastic resonance, deep artificial neural networks, dorsal cochlear nucleus, tinnitus mechanisms, Zwicker tone

## Abstract

Noise is generally considered to harm information processing performance. However, in the context of stochastic resonance, noise has been shown to improve signal detection of weak sub- threshold signals, and it has been proposed that the brain might actively exploit this phenomenon. Especially within the auditory system, recent studies suggest that intrinsic noise plays a key role in signal processing and might even correspond to increased spontaneous neuronal firing rates observed in early processing stages of the auditory brain stem and cortex after hearing loss. Here we present a computational model of the auditory pathway based on a deep neural network, trained on speech recognition. We simulate different levels of hearing loss and investigate the effect of intrinsic noise. Remarkably, speech recognition after hearing loss actually improves with additional intrinsic noise. This surprising result indicates that intrinsic noise might not only play a crucial role in human auditory processing, but might even be beneficial for contemporary machine learning approaches.

## Introduction

The term *noise* usually describes undesirable disturbances or fluctuations, and is considered to be the *“fundamental enemy”* (McDonnell and Abbott, 2009) for communication and error-free information transmission and processing in engineering. However, a vast and still increasing number of publications demonstrate the various benefits of noise for signal detection and processing, among which the most important phenomena are called stochastic resonance (McDonnell and Abbott, 2009), coherence resonance (Pikovsky and Kurths, 1997), and recurrence resonance (Krauss et al., 2019a).

The term stochastic resonance (SR), first introduced by Benzi et al. (1981), refers to a processing principle in which signals that would otherwise be sub-threshold for a given sensor can be detected by adding a random signal of appropriate intensity to the sensor input (Benzi et al., 1981; Gammaitoni et al., 1998; Moss et al., 2004). SR occurs ubiquitously in nature and covers a broad spectrum of systems in physical and biological contexts (Wiesenfeld and Moss, 1995; McDonnell and Abbott, 2009). Especially in neuroscience, it has been demonstrated to play an essential role in a vast number of different systems (Douglass et al., 1993; Collins et al., 1996; Gluckman et al., 1996; Nozaki et al., 1999; Usher and Feingold, 2000; Ward et al., 2002; Kosko and Mitaim, 2003; Aihara et al., 2008; Faisal et al., 2008). Also, it has already been proposed that spontaneous random activity, i.e., noise, may increase information transmission *via* SR in the auditory brain stem (Mino, 2014).

In self-adaptive signal detection systems based on SR, the optimal noise intensity is continuously adjusted *via* a feedback loop so that the system response remains optimal in terms of information throughput, even if the characteristics and statistics of the input signal change. The term adaptive SR was coined for this processing principle (Mitaim and Kosko, 1998, 2004; Wenning and Obermayer, 2003). In a previous study we demonstrated that the auto-correlation of the sensor output, a quantity always accessible and easy to analyze by neural networks, can be used to quantify and hence maximize information transmission even for unknown and variable input signals (Krauss et al., 2017).

In further studies we demonstrated theoretically and empirically that adaptive SR based on output auto-correlations might be a major processing principle of the auditory system that serves to partially compensate for acute or chronic hearing loss, e.g., due to cochlear damage (Krauss et al., 2016, 2018, 2019b; Gollnast et al., 2017; Krauss and Tziridis, 2021; Schilling et al., 2021d). Here, the noise required for SR would correspond to increased spontaneous neuronal firing rates in early processing stages of the auditory brain stem and cortex, and would be perceived as a phantom perception. Remarkably, this phenomenon has frequently been observed in animal models and in humans with subjective tinnitus (Wang et al., 1997; Ahlf et al., 2012; Tziridis et al., 2015; Wu et al., 2016), which in turn is assumed to be virtually always caused by some kind of apparent (Heller, 2003; Nelson and Chen, 2004; König et al., 2006; Shore et al., 2016) or hidden hearing loss (Schaette and McAlpine, 2011; Liberman and Liberman, 2015). From this point of view, phantom perceptions like tinnitus seem to be a side effect of an adaptive mechanism within the auditory system whose primary purpose is to compensate for reduced input through continuous optimization of information transmission (Krauss et al., 2016, 2018, 2019b; Krauss and Tziridis, 2021; Schilling et al., 2021d). This adaptive mechanisms can also be investigated by simulating a hearing loss. Thus, the presentation of a white noise stimulus with a spectral notch, which leads to reduced input in a certain frequency range, leads to better hearing thresholds within this frequency range on the one hand (Wiegrebe et al., 1996; Krauss and Tziridis, 2021) and causes an auditory phantom perception—the so called Zwicker tone (Zwicker, 1964; Parra and Pearlmutter, 2007)—after noise offset, on the other hand.

The dorsal cochlear nucleus (DCN) was shown to be the earliest processing stage, where decreased cochlear input, due to acoustic trauma induced hair cell loss and synaptopathy (Liberman et al., 2016; Tziridis et al., 2021), results in increased spontaneous firing rates (Kaltenbach et al., 1998; Kaltenbach and Afman, 2000; Zacharek et al., 2002; Wu et al., 2016). Interestingly, the amount of this increase in spontaneous activity, i.e., neural hyperactivity, is correlated with the strength of the behavioral signs of tinnitus in animal models (Brozoski et al., 2002; Kaltenbach et al., 2004). Furthermore, the hyperactivity is localized exclusively in those regions of the DCN that are innervated by the damaged parts of the cochlea (Kaltenbach et al., 2002). Gao et al. (2016) recently described changes in DCN fusiform cell spontaneous activity after noise exposure that supports the proposed SR mechanism. In particular, the time course of spontaneous rate changes shows an almost complete loss of spontaneous activity immediately after loud sound exposure (as no SR is needed due to stimulation that is well above threshold), followed by an overcompensation of spontaneous rates to levels well above pre-exposition rates since SR is now needed to compensate for acute hearing loss (Gao et al., 2016). It is well-known that the DCN receives not only auditory input from the cochlea, but also from the somatosensory system (Young et al., 1995; Nelken and Young, 1996; Ryugo et al., 2003; Shore and Zhou, 2006; Koehler and Shore, 2013; Wu et al., 2016; Ansorge et al., 2021; Niven and Scott, 2021), and that noise trauma alters long-term somatosensory-auditory processing in the DCN (Dehmel et al., 2012), i.e., somatosensory projections are up-regulated after deafness (Zeng et al., 2012).

In self-adaptive signal detection systems based on SR, the optimal noise level is continuously adjusted so that the system response in terms of information throughput remains optimal, even if the properties of the input signal change. The term adaptive SR was coined for this processing principle (Mitaim and Kosko, 1998, 2004). An objective function for quantifying information content is the mutual information be- tween the sensor input and the output (Shannon, 1948), which is often used in theoretical approaches (Levin and Miller, 1996; Mitaim and Kosko, 2004; Moss et al., 2004). The choice of mutual information is obvious, since the basic purpose of each sensor is to transmit information to a subsequent information processing system. It has already been shown that the mutual information has a maximum as a function of the noise intensity, which indicates the optimal noise level that has to be added to the input signal in order to achieve optimal information transmission by SR (Moss et al., 2004). A fundamental disadvantage of the mutual information, however, is the impossibility of calculating it in every application of adaptive SR if the signal to be recognized is unknown (Krauss et al., 2017). Even if the underlying signal is known, the use of mutual information in the context of neural network architectures seems to be rather impractical, since its calculation requires the evaluation of probability distributions, logarithms, products and fractions, i.e., operations difficult to implement in neural networks. In an earlier work (Krauss et al., 2017) we were able to show that this fundamental disadvantage can be overcome by another objective function, namely the autocorrelation of the sensor response. Both, the mutual information and the autocorrelation peak at the same noise level. Hence, maximization of the output autocorrelation leads to similar or even identical estimates of the optimal noise intensities for SR as the mutual information, but with the decisive advantage that no knowledge of the input signal is required (Krauss et al., 2017). In contrast to mutual information, the evaluation of autocorrelation functions in neural networks can easily be implemented using delay lines and coincidence detectors (Licklider, 1951). Remarkably, a cerebellar-like neuronal architecture resembling such delay-lines is known to exist in the DCN (Osen et al., 1988; Hackney et al., 1990; Nelken and Young, 1994; Oertel and Young, 2004; Baizer et al., 2012). Therefore, we previously proposed the possibility that the neural noise for SR is injected into the auditory system *via* somatosensory projections to the DCN (Krauss et al., 2016, 2018, 2019b; Krauss and Tziridis, 2021; Schilling et al., 2021d,^2022^). The idea that central noise plays a key role in auditory processing has recently gained increasing popularity (Zeng, 2013, 2020; Koops and Eggermont, 2021) and is supported by various findings. For instance, it is well-known, that jaw movements lead to a modulation of subjective tinnitus loudness (Pinchoff et al., 1998). This may easily be explained within our framework, as jaw movements alter somatosensory input to the DCN. Since this somatosensory input corresponds to the noise required for SR, auditory input to the DCN is modulated through this mechanism, and the altered noise level is then perceived as modulated tinnitus (Krauss et al., 2016, 2018, 2019b; Schilling et al., 2021d). Along the same line, one may explain why both, the temporomandibular joint syndrome and whiplash, frequently cause so called somatic tinnitus (Levine, 1999). Another example is the finding of Tang and Trussell (2015, 2017), who demonstrated that somatosensory input and hence tinnitus sensation may also be modified by serotonergic regulation of excitability of principal cells in the DCN. In addition, DCN responses to somatosensory stimulation are enhanced after noise-induced hearing loss (Shore et al., 2008; Shore, 2011). Finally, and most remarkable, electro-tactile stimulation of finger tips, i.e., increased somatosensory input, significantly improves both, melody recognition (Huang et al., 2019) and speech recognition (Huang et al., 2017) in patients with cochlear implants.

However, while we propose the DCN to be the place where auditory input from the cochlea is integrated with neural noise from the somatosensory system, we cannot rule out that SR rather occurs in the ventral cochlear nucleus (VCN) instead (see “Discussion” section).

**FIGURE 1:**
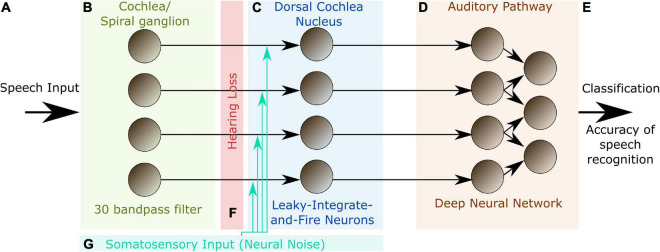
Model layout. The complete model consists of three different modules representing different stages of the auditory pathway in the human brain. The input to the model are single words encoded as wave files with a sampling rate of 44.1 kHz and 1 s duration **(A)**. The cochlea and the spiral ganglion are modeled as an array of 30 band-pass filters **(B)**. The continuous output signal of **(B)** serves as input to 30 leaky integrate-and-fire-neurons representing the DCN **(C)**. The spike-train output of the DCN model is down sampled and serves as input for a deep neural network that is trained with error backpropagation on the classification of 207 different German words **(D)**. The classification accuracy serves as a proxy for speech recognition **(E)**. In order to investigate the effect of a particular hearing loss, the cochlea output amplitude is decreased by a certain factor independently for all frequency channels **(F)**. White noise representing somatosensory input to the DCN can be added independently to the input of the different leaky-integrate-and-fire-neurons (LIF, **G**).

In order to further support the hypothesis that SR plays a key role in auditory processing, we here present a hybrid computational model of the auditory pathway, trained on speech recognition. An overview of the model layout is provided in Figure 1.

The model is not intended to be a fine-grained model of the complete auditory pathway with exhaustive biological detail, but is rather used to demonstrate, analyze and interpret the basic principles of information processing in the auditory system. Thus, we abstracted from most biological details and constructed a coarse-grained model of the cochlea, which does not cover the full potential of cochlear information processing compared to more fine-grained implementations as introduced e.g., by Carney (1993, 2021), Sumner et al. (2002), James et al. (2018), and Verhulst et al. (2018). Thus, Carney and co-workers simulate the cochlea as narrow-band filters but applied a feed-back loop changing the parameters of this filters with intensity (Carney, 1993). Sumner and coworkers model the molecular mechanisms including the distribution of calcium ions and neurotransmitter release (Sumner et al., 2002) in the cochlea and Verhulst and coworkers map their model on existing neurophysiological recordings of human subjects and animals (Verhulst et al., 2018).

In our approach, also the DCN circuitry is not modeled in all detail, but only as a one-layered structure of leaky-integrate-and-fire neurons, which are not interconnected. The aim of our implementation is not to understand the whole auditory pathway in detail, which would be far to ambitious, but to find out if SR could have a significant effect on speech perception. Thus, it is not the aim of the study to analyze the auditory system on an implementational level (see Marrs’ level of analysis; Marr and Poggio, 1979), but to explain the algorithmic level (Krauss and Schilling, 2020; Schilling et al., 2022).

The output of the DCN is fed to a deep neural network trained on word recognition. The deep neural network can be interpreted as a surrogate for all remaining stages of the auditory pathway beyond the DCN up to the auditory cortex. However, it may also be regarded as a tool to quantify the information content of the DCN output. The deep neural network was trained once on a training data set and kept stable for the experiments.

**FIGURE 2:**
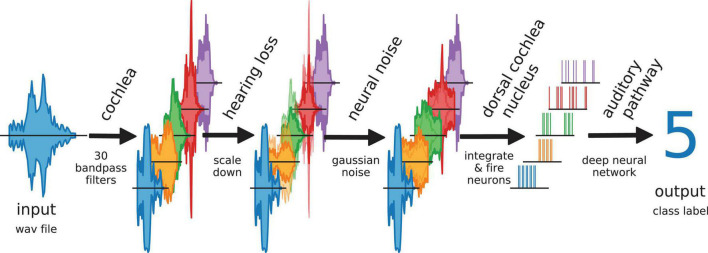
Data flow in auditory pathway model. The scheme shows how the speech data is processed within the model. The cochlea splits the signal *via* 30 bandpass filters. The bandpass filtered data is scaled down to simulate a hearing loss. The hearing loss affects only channels within the speech relevant frequency range (orange, green, red). The other frequency channels are unchanged. Neural noise is added to investigate the effect of stochastic resonance (only in hearing impaired channels). The DCN is simulated as 30 LIF neurons. Each LIF neuron represents a complete biological neuron population. The spike data is down-sampled and fed to the deep neural network.

Furthermore, we simulate different levels of hearing loss (cochlear damage) and compare the resulting word recognition accuracies for with the accuracy of the non-disturbed model (i.e., without simulated hearing loss). Subsequently, we add intrinsic noise of different intensities to the model. The overall data flow in our model is depicted in Figure 2.

As expected and shown in various experimental studies with human subjects (Lorenzi et al., 2006; Zeng and Liu, 2006) we find in our model that speech recognition accuracy decreases systematically with increasing hearing loss (Zeng and Djalilian, 2010). In the case of additional intrinsic noise, we find SR-like behavior for all levels of hearing loss: depending on the intensity of the noise, accuracy first increases, reaches a peak, and finally decreases again. This means that speech recognition after hearing loss may indeed be improved by our proposed mechanism. A simple increase of the spontaneous activity of the DCN neurons did not lead to an increased speech recognition, which indicates that indeed SR causes the increase in word recognition accuracy. This intriguing result indicates, that SR indeed plays a crucial role in auditory processing, and might even be beneficial for contemporary machine learning approaches.

## Results

### Dorsal Cochlear Nucleus Model Neurons Show Phase Coupling Below 4 kHz

**FIGURE 3:**
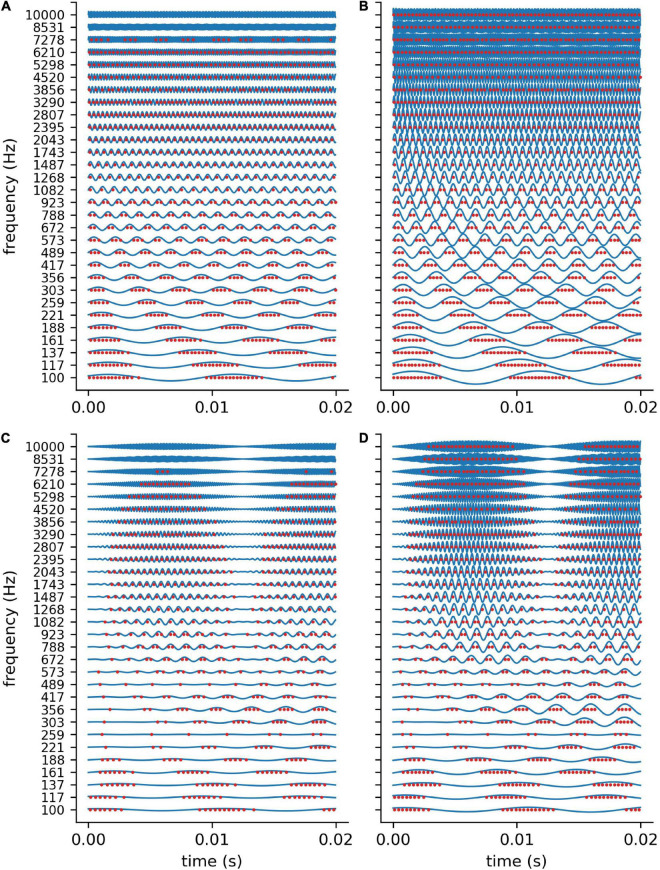
DCN model response to sine waves. Shown are the spiking outputs of the LIF neurons for sine input with two different constant amplitudes (**A**: 0.001, **B**: 0.002), and two different amplitude modulations **(C,D)**. For lower amplitudes **(A)** and higher frequencies the LIF neurons do not spike at all, whereas for higher amplitudes a rate code can be observed as the neurons’ maximum spiking rate is limited due to the refractory period. The parameters of the LIF neurons are chosen so that there is phase coupling in the frequency range which is relevant for speech perception.

In order to validate our DCN model, we investigate the spike train output of the 30 leaky integrate- and-fire (LIF) neurons for different sine wave inputs (Figure 3). As described in detail in “Methods” section, the parameters of the LIF neurons are chosen so that the refractory time (0.25 ms) of the neurons does not allow for firing rates above 4 kHz. This is much more than the maximum spiking rate of a biological neuron (400 Hz) (Nizami, 2002). However, the recruitment of several neurons to increase the frequency range in which phase coupling is possible is a core concept within the dorsal cochlear nucleus (Langner, 1988). Thus, in our model 1 simulated LIF neuron represents approximately 10 biological neurons, having individual refractory times above 1 ms.

We find that for stimulus frequencies above 4 kHz and amplitudes of 0.001 the LIF neurons do not spike at all (Figure 3A). In contrast, for a larger amplitude of 0.002, a rate coding without phase coupling can be observed (Figure 3B). Furthermore, we find that the LIF neurons are sensitive to amplitude modulations also in the frequency range above 4 kHz (Figures 3C,D). Thus, our DCN neurons are designed so that they allow for phase coupling in the frequency range crucial for speech comprehension, as is known from the human auditory system.

### Word Processing From Cochlea to Dorsal Cochlear Nucleus

**FIGURE 4:**
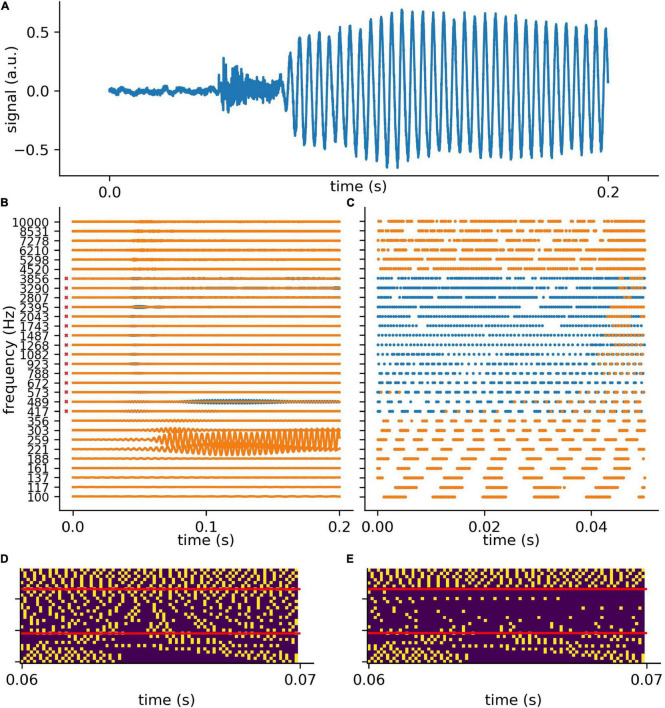
Exemplary processing of a word in cochlea and DCN model. **(A)** The first 0.2 s of audio data of the German word “die” (the). **(B)** The 30 frequency components (blue without hearing loss, orange with hearing loss) after the first part of the model, which represents the cochlea and the spiral-ganglion (Figure 1A). A virtual hearing loss is applied by weakening the signal at a certain frequency range (e.g., 400 Hz–4 kHz, −30 dB). The bandpass filtered signal (matrix of 30 frequency channels and *f*_*s*_ × signal duration) is fed to the LIF neurons (refractory time: ≈ 0.25 *ms*) and spike trains **(C)** are generated. These spike trains are down-sampled by a factor of 5 and fed to the deep neural network **(D)**. **(E)** The same signal (of panel **D**) with added hearing loss of 30 dB in the frequency range 400 Hz–4 kHz being the speech relevant range.

In analogy to the auditory system, the complex auditory stimuli representing spoken words (Figure 4A) are transformed in the cochlea into continuous signals in a number of different frequency channels, in our model 30. However, the cochlea does not perform a simple Fourier transform, but rather splits the signal into multiple band pass filtered signals, thereby preserving the complete phase information (Figure 4B). For the purpose of simplicity, in the context of our model we assume that the auditory nerve fibers directly transmit this analog signal to the DCN, which is regarded to be a special feature of the auditory system (Kandel et al., 2000; Young and Davis, 2002).

**FIGURE 5:**
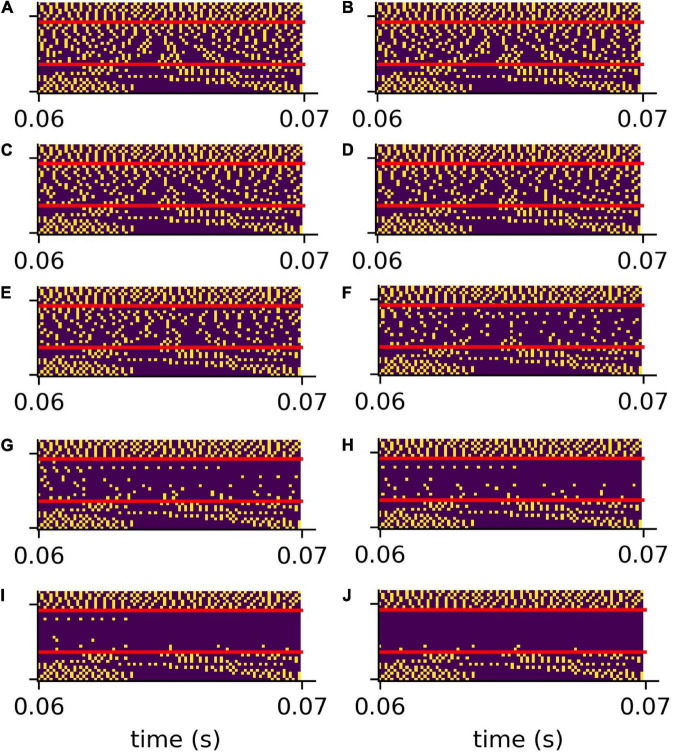
Compressed spike patterns with added hearing loss. The figure shows the down-sampled spike patterns of the same word as shown in Figure 4. The speech relevant frequency range (400 Hz–4 kHz) is artificially weakened (hearing loss). Panles **(A–J)** refer to hearing losses 0–45 dB.

The analog signals are then further transformed into spike train patterns in the DCN (Figure 4C). Thus, each spoken word is represented as a unique spiking pattern with a dimensionality of 30 × *N*, where 30 corresponds to the number of frequency channels and *N* is the sampling rate in Hz times the word length in seconds. Note that we down-sampled these matrices by a factor of five from 44,100 to 8,200 Hz for deep learning (Figure 4D). This does not affect the phase coupling information in the speech relevant frequency range. In order to analyze speech processing in an impaired auditory system, we simulated a hearing loss in the speech relevant frequency range (400 Hz–4 kHz) by decreasing the cochlea output amplitudes by a certain factor. The weakened cochlea outputs and the resulting modified DCN spike train outputs are shown in Figures 4B,C, where orange corresponds to an exemplary hearing loss of 30 dB, and blue corresponds to the undisturbed signals, i.e., without hearing loss. The corresponding down sampled spike pattern matrices used as test data for the deep neural network, are shown in Figure 4D (without hearing loss) and in Figure 4E (with 30 dB hearing loss). We provide an exemplary overview of the effect of different hearing losses from 0 to 45 dB on the spike pattern matrices in Figure 5.

### Intrinsic Noise Partially Restores Spike Patterns After Simulated Hearing Loss

**FIGURE 6:**
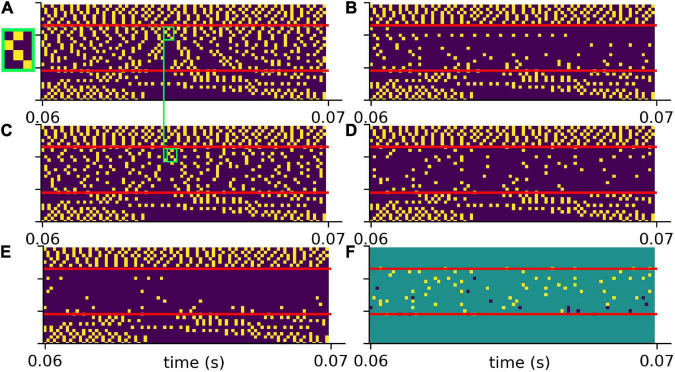
Effect of intrinsic noise on the DCN output patterns. **(A)** Spiking without HL (same as in Figure 5A). **(B)** Spiking with a HL of 30 dB (same as Figure 5G). **(C)** Spiking activity with HL and intrinsic noise of optimal intensity. Additional white noise increases spiking activity. **(D)** Point-to-point comparison of spiking patterns for no HL and with HL and intrinsic noise. Shown are only spikes that occur in both cases, i.e., that are not affected by HL or that are correctly restored by noise. **(E)** Point-to-point comparison of spiking patterns for no HL with HL and without intrinsic noise. Shown are only spikes that occur in both cases, i.e., that are not affected by HL. **(F)** Intrinsic noise of optimal intensity not only restores spikes correctly (yellow), but also introduces false positive spikes (dark blue). Intrinsic noise restores spatio-temporal spiking patterns correctly, yet with some temporal shift (green boxes in panels **A,C**, zoom of spike pattern in green box).

To test the putative beneficial effect of intrinsic noise in case of hearing loss, we analyzed spiking patterns generated with and without intrinsic noise and compared them with the corresponding undisturbed patterns (Figure 6). In Figure 6A a sample spike pattern in case of no hearing loss is shown as reference. As expected, a simulated hearing loss of 30 dB in the frequency range of 400 Hz to 4 kHz leads to a decreased spiking activity (Figure 6B), which can be partially restored by the addition of intrinsic noise with optimal intensity (Figure 6C).

A point-to-point comparison of the spikes resulting from the undisturbed system (no hearing loss) with the spikes resulting from hearing loss and additional intrinsic noise, demonstrates that there is indeed some improvement. In Figure 6D only those spikes are shown that occur in both mentioned cases. In contrast, there are less spikes resulting from hearing loss without intrinsic noise (Figure 6E). Further analysis yield that intrinsic noise not only restores spikes correctly (Figure 6F, yellow), but also introduces false positive spikes (Figure 6F, blue). However, a direct point-to-point comparison of spike patterns does not fully capture the benefit of intrinsic noise. As shown in Figures 6A,C (green boxes), intrinsic noise even restores larger spatio-temporal spiking patterns correctly, yet with some temporal shift.

### Intrinsic Noise Improves Accuracy for Speech Recognition After Simulated Hearing Loss

We also analyzed the effect of intrinsic noise on speech recognition accuracy in case of hearing loss in different scenarios. Using our custom-made data set, we investigated hearing loss in two different frequency ranges. Furthermore, using the free spoken digit data set (FSDD) data set, we investigated hearing loss using two different neural networks. In all cases, we find that intrinsic noise of appropriate intensity improves accuracy for speech recognition after simulated hearing loss. Note that the weights of the deep neural network are kept constant for all further analyses. Thus, the relative accuracy is normalized to the original test accuracy (0.37) of the undisturbed network.

### Custom-Made Data Set and Hearing Loss in the Frequency Range of 400 Hz–4 kHz

**FIGURE 7:**
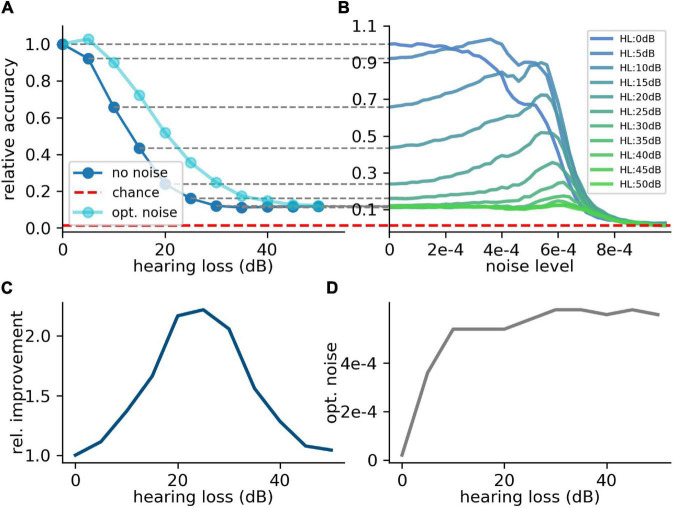
Effect of SR on speech recognition. **(A)** The curve shows the relative accuracy of the trained neural network as a function of the hearing loss (red dashed line: chance level; $\frac{\left(1/207\right)}{\text{max}\,\text{accuracy}}$). The hearing loss (5–50 dB, 5 dB steps, frequency range of HL: 400 Hz–4,000 Hz) was implemented in the test data set and propagated through the pre-trained network. Thus, the cochlea output was multiplied with an attenuation factor ($10^{\frac{-\mathrm{HL}}{20}}$). This output was then transformed using the integrate-and-fire neurons and fed in the neural network. **(B)** Relative accuracy as a function of the applied noise level for different hearing losses. Resonance curves with one global maximum at a certain noise level > 0 could be shown. **(C)** Best relative improvement as a function of the hearing loss. **(D)** Optimal noise level as a function of the hearing loss.

For the first scenario, we used a convolutional neural network (Table 1) trained on our custom-made data set. After training, we simulated a hearing loss in the frequency range of 400 Hz to 4 kHz which is known to be crucial for speech comprehension in humans (Fox, 2006). The effect of improved or decreased speech comprehension is quantified by the classification accuracy of the words (test accuracy). The classification accuracy as a function of the hearing loss has a biologically plausible sigmoid shape (Figure 7A dark blue curve). The test accuracies as a function of the added noise for different hearing losses show a clear resonance curve with a global maximum (Figure 7B). For a hearing loss of about 20 dB, the relative improvement of speech comprehension is more than doubled (Figure 7C). Furthermore, it can be shown that the optimal noise level correlates with the hearing loss (Figure 7D). This effect is plausible as for a weaker signal a higher noise amplitude is needed to lift the signal over the threshold of the LIF neurons. In summary, it can be stated that the addition of noise can lead to an improved speech comprehension for all hearing losses. This fact can be seen in Figure 7A, where the cyan curve shows the test accuracy as a function of the hearing loss with the ideal amount of added Gaussian noise.

**TABLE 1:** Exact parameters of the used deep convolutional network (main analysis).

Layer	Type	Input-output-dim	Activation	Characteristics
1	Convolution 2D	30, 8,820, 1; 1, 8,791, 128	ReLu	
2	Reshape	1, 8,791, 128; 8,791, 128		
3	Convolution 1D	8,791, 128; 8,782, 128	ReLu	
4	MaxPooling 1D	8,782, 128; 4,391, 128		Pool size: 2
5	DropOut	4,391, 128; 4,391, 128		Dropout: 0.5
6	Convolution 1D	4,391, 128; 4,391, 128	ReLu	
7	Convolution 1D	4,391, 128; 4,390, 128	ReLu	
8	MaxPooling 1D	4,390, 128; 2,195, 128		Pool size: 2
9	DropOut	2,195, 128; 2,195, 128		Dropout: 0.5
10	Flatten	2,195, 128; 280,960		
11	Dense	280,960; 150	ReLu	
12	Dense	150; 50	ReLu	
13	Dense	50; 207	Softmax	

### Custom-Made Data Set and Hearing Loss in the Frequency Range Above 4 kHz

**FIGURE 8:**
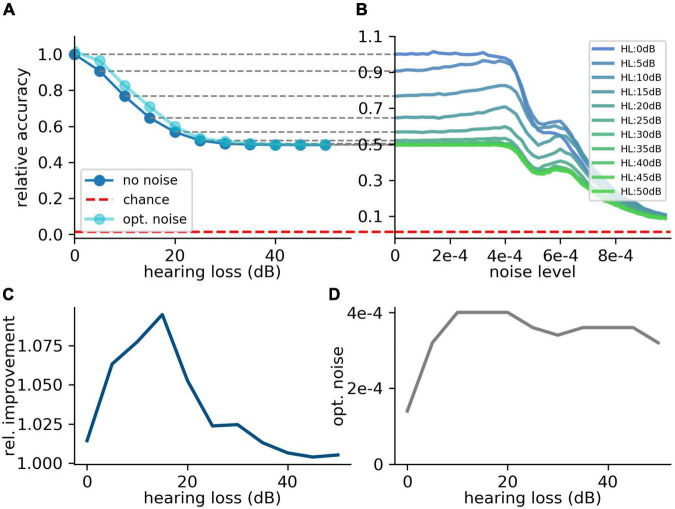
Effect of SR on speech recognition (high frequency hearing loss). Same analysis as shown in Figure 7 for high frequency hearing loss. **(A)** The plots show the relative accuracy of the trained neural network as a function of the hearing loss (red dashed line: chance level). The high frequency hearing loss lead to different effects (10–50 dB, 10 dB steps, frequency range of HL: above 4,000 Hz). **(B)** The relative accuracy as a function of the noise has no clear maximum above the value for no added noise (nearly no SR). Furthermore, a second local maximum occurs. **(C)** The best relative improvement does not significantly increase over 10%. **(D)** Optimal noise level as a function of hearing loss shows similar behavior as for the hearing loss in the speech relevant frequency range (cf. Figure 7).

Since many people suffer from hearing losses in the high frequency range (Ciorba et al., 2011). In the next step, the stochastic resonance effect is analyzed for a high frequency range hearing loss starting at a frequency of 4 kHz. It can be shown that the high frequency loss does not affect the speech comprehension abilities in the same manner as hearing losses in the critical frequency range between 400 Hz and 4 kHz (Figure 8A). The relative accuracy does not drop below a value of 50%. Thus, the effect of stochastic resonance is also reduced (Figure 8B), which means a maximal relative improvement of approximately 10% (Figures 8C,D). Furthermore, there is no real resonance curve with one maximum at a certain noise frequency but a second maximum at a higher noise level (Figure 8B). To put it in a nutshell, we can state that the addition of noise can lead to a significant improvement of speech recognition.

### Custom-Made Data Set and Hearing Loss With Non-linearity in the Frequency Range of 400 Hz to 4 kHz

**FIGURE 9:**
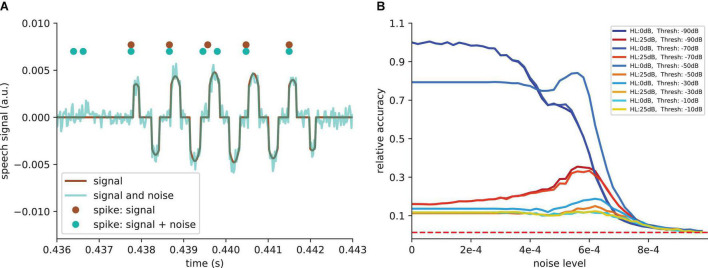
SR effect with additional threshold. **(A)** Example of signal after simulated cochlea for one frequency channel and an additional threshold of −50 dB (−50 dB means that all values above $-10^{\frac{-50}{20}}$ and below $10^{\frac{-50}{20}}$≈ 0.003 are set to zero). This threshold is introduced to show that the SR effect also works when hearing loss leads to a real information loss. The noise leads to a signal enhancement (cyan curve). Thus, the signal causes more spiking in the DCN (cyan dots compared to brown dots). **(B)** Relative accuracy as a function of the amplitude of the added noise. SR resonance partly restores the accuracy. For very high thresholds, where main parts of the signals are deleted, the SR does not restore the accuracy.

So far, we simulated linear hearing loss in the model cochlea. However, it is known that different damages to the cochlea or the synapses from the cochlea to the cochlear nuclei yield to different degrees of non-linearity in hearing loss. Therefore, we also tested our model with an additional threshold of −50 dB, i.e., all values above $-10^{\frac{-50}{20}}$ and below $10^{\frac{-50}{20}}$≈ 0.003 are set to zero. Also in the case of an additional hard threshold, leading to real information loss, the SR effect still works. The added noise leads to a signal enhancement. Thus, the signal causes more spiking in the DCN (Figure 9A). Consequently, the relative speech recognition accuracy is partly restored by SR (Figure 9B).

### Free Spoken Digit Data Set Data Set and Hearing Loss in the Frequency Range Above 400 Hz

**FIGURE 10:**
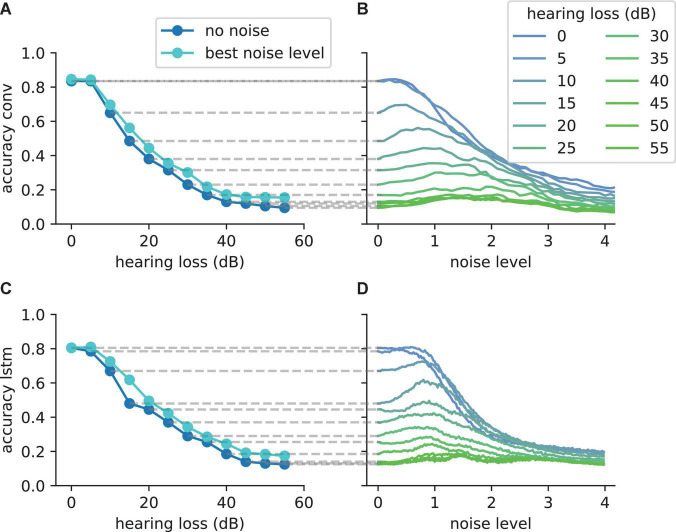
The SR resonance effect in different network architectures using the FSDD data set. **(A)** The plot shows the test accuracy as a function of the applied hearing loss for a deep convolutional network architecture (dark blue, starting at 400 Hz, exact network architecture shown in Table 2) trained on English words (digits: 0–9). The impaired speech comprehension by the hearing loss can be partly compensated by adding Gaussian noise (stochastic resonance). The cyan curve shows the improvement of speech comprehension for the optimal noise level (maxima values in panel **B**). **(B)** Test accuracy for different hearing losses (shades of blue) as a function of the added noise. The maxima show that SR can help to restore speech comprehension. **(C)** Similar analysis as shown in panel **(A)** for a two layer LSTM network (exact network architecture shown in Table 3); **(D)** Similar analysis as shown in panel **(B)** for the LSTM architecture. The improvement of speech perception in impaired systems (hearing loss) is a universal principle and does not depend on the used neural network.

In order to further demonstrate that the reported results are not limited to a certain data set, natural language or neural network architecture, we repeated our analyses using two further neural networks, an alternative convolutional neural network architecture (Table 2) and a network with Long-Short-Term-Memories (Table 3), both trained and tested on English language, i.e., the FSDD data set (Figure 10). A hearing loss in the critical frequency range for speech comprehension leads to a decrease in the classification accuracy (10a for the convolutional network and 10c for the Long- Short-Term-Memory network). Furthermore, the stochastic resonance effect in terms of a clear resonance curve with one maximum can be observed (Figures 10B,D).

**TABLE 2:** Exact parameters of the used deep convolutional network (FSDD data set).

Layer	Type	Input-output-dim	Activation	Characteristics
1	Convolution 2D	9,131, 30, 1; 9,102, 1, 32	ReLu	
2	MaxPooling 2D	9,102, 1, 32; 4,551, 1, 32		Pool size: (2, 1)
3	DropOut	4,551, 1, 32; 4,551, 1, 32		Droupout: 0.2
4	Convolution 2D	4,551, 1, 32; 4,547, 1, 64	ReLu	
5	MaxPooling 2D	4,547, 1, 64; 2,273, 1, 64		Pool size: (2, 1)
6	DropOut	2,273, 1, 64; 2,273, 1, 64		Dropout: 0.2
7	Convolution 2D	2,273, 1, 64; 2,272, 1, 32	ReLu	
8	MaxPooling 2D	2,272, 1, 32; 1,136, 1, 32		Pool size: (2, 1)
9	DropOut	1,136, 1, 32; 1,136, 1, 32		Dropout: 0.2
10	Flatten	1,136, 1, 32; 36,352		
11	Dense	36,352; 400	ReLu	
12	DropOut	400; 400		Dropout: 0.2
13	Dense	400; 50	ReLu	
14	Dense	50; 10	Softmax	

**TABLE 3:** Exact parameters of the used LSTM network (FSDD data set).

Layer	Type	Input-output-dim	Activation	Characteristics
1	GroupToBatches	9,000, 30; 45, 6,000		
2	LSTM	45, 6,000; 45, 200	tanh	
3	DropOut	45, 200; 45, 200		Dropout: 0.5
4	LSTM	45, 200; 45, 100	tanh	
5	DropOut	45, 100; 45, 100		Dropout: 0.5
6	TimeDistributed Dense	45, 100; 45, 100		
7	DropOut	45, 100; 45, 100		Dropout: 0.5
8	TimeDistributed Dense	45, 100; 45, 10		

## Methods

### Computational Resources

The simulations were run on a desktop computer equipped with an i9 extreme processor (Intel) with 10 calculation cores. Furthermore, the machine learning was run on the same computer on two Nvidia Titan XP graphical processor units. To test the validity of our calculations the simulations were performed on two different code bases. The main results based on our own speech data set are mainly based on Numpy (Walt et al., 2011) and SciPy (Jones et al., 2001) calculations. The convolutional network was implemented in Keras (Chollet, 2018) with Tensorflow (Abadi et al., 2016) back-end. All main results were confirmed by analyzing a standard speech data set—the so called Jakobovski free spoken digit data set (FSDD) (Jackson et al., 2018), containing spoken numbers from 0 to 9 in English language in accordance to the MNIST data set with written digits in this range (LeCun et al., 1998). This was done using a completely new code base exclusively build of KERAS layers. Thus, a custom-made KERAS layer implemented as sinc FIR filters for the cochlea layer as well as the leaky-integrate-and-fire neurons were implemented. All plots were created using the Matplotlib Python library (Hunter, 2007) and plots were arranged using the pylustrator (Gerum, 2020).

### Layout of the Computational Model and General Approach

The model comprises three modules (Figure 1): (1) an artificial cochlea modeled as an array of band-pass filters, (2) a model of the dorsal cochlear nucleus (DCN), implemented as an array of leaky integrate-and-fire (LIF) neurons, and (3) a deep neural network, that represents all further processing stages beyond the DCN up to the auditory cortex and higher, language associated, cortex areas.

The input to the model are single words of spoken language encoded as wave files with a sampling rate of 44.1 kHz and 1 s duration (Figure 1A). These wave files represent the acoustic input of speech to the auditory system, and are processed in the first module of the model representing the cochlea and the spiral ganglion (Figure 1B). Like in previously published models (Moore and Glasberg, 1983; Houser et al., 2001; Sayles and Winter, 2010), this module is implemented as an array of rectangular band-pass filters. In order to limit the total computation time, we restricted our model to 30 band-pass filters, instead of the actual amount of approximately 3,500 inner hair cells in the human cochlea (Nadol, 1988). According to the physiology of the cochlea (Russell and Nilsen, 1997), the center frequencies of the band-pass filters are chosen such that they cover the frequency range from 100 Hz to 10 kHz in logarithmic steps.

The continuous multi-channel output of the band-pass filter array serves as input to an array of 30 LIF neurons (Burkitt, 2006) representing the DCN (Figure 1C). We here applied a one-to-one mapping from band-pass filters to model neurons, i.e., we do not explicitly account for putative cross-talk between neighboring frequency channels. However, since both the cochlea and the DCN model only consist of 30 different frequency channels, each of these channels may be regarded as an already coarse grained version of approximately 100 different frequency channels that exist in the human auditory system. Thus, eventual cross-talk is implicitly implemented in our model within each of the 30 modeled channels. The output of our DCN model comprises the spike trains of the 30 LIF neurons. Note that, in our DCN model, a single LIF neuron represents approximately 10 biological neurons processing the same frequency channel (Kandel et al., 2000).

In our cochlea and DCN model, the outputs of the band-pass filters and the membrane potentials of the LIF neurons change with the same rate (44.1 kHz) as the wave file input. However, the LIF neurons spike at lower average rates, due to their refractory period. It is therefore possible to down-sample this sparse output spike train, thereby reducing the data volume for the subsequent deep neural network. In order to preserve enough temporal information for phase coding, we down-sample the DCN output only by a factor of five, so that the 44,100 momentary amplitudes of the input wave file per second are finally transformed into a binary 30 × 8,820 matrix.

These binary matrices serve as training input for the deep neural network, representing all further processing stages beyond the DCN up to the auditory cortex and higher, language associated, cortex areas. The neural network consists of four convolutional layers and three fully connected layers, and is trained with error backpropagation on the classification of 207 different German words (Figure 1D). The resulting classification accuracy of the trained network serves as a proxy for speech recognition (Figure 1E).

In order to simulate a particular hearing loss, the output amplitudes of the cochlea model are decreased by a certain factor, independently for the different frequency channels (Figure 1F). Subsequently, these modified cochlea outputs are further processed in the LIF neurons, finally resulting in a new binary matrix for each word for a particular hearing loss. These new matrices then serve as test data for the previously trained deep neural network, yielding a new classification accuracy. By comparing the reference test accuracy (without any hearing loss) with the new test accuracy, the effect of a particular hearing loss on speech recognition was estimated.

Optionally, Gaussian noise with zero mean and a certain standard deviation, representing somatosensory input to the DCN, was added independently to the input of each LIF neuron (Figure 1G). Here, the standard deviation corresponds to the noise intensity. As described before, again this finally results in a new binary matrix for each wave file, yet corresponding to a particular hearing loss and, in addition, also to a particular set of frequency channel specific noise intensities. Again, all these new matrices serve as test data for the deep neural network. By comparing the reference test accuracy (without any hearing loss and noise) with the new test accuracy, the effect of particular noise intensities on speech recognition with a certain hearing loss was estimated. A sketch of the complete data flow in case of certain hearing loss and additional noise is depicted in Figure 2.

### Simplified Model of the Cochlea

The cochlea is simulated as 30 Butterworth bandpass filters (3rd order) with no overlapping bands. These 30 bandpass filters are a simplification of the more than 3,000 inner hair cells of the human cochlea (Dallos, 1992). In contrast to other complex cochlea models (Tan and Carney, 2003; Chambers et al., 2019), this simplification of the dynamics of the inner hair cells was chosen to derive basic principles and to increase interpretability. The center frequencies (of the bandpass filters) are between 100 Hz (minfreq.) and 10 kHz (maxfreq.) including the complete frequency range needed for speech comprehension. The center frequencies are chosen to grow exponentially [centerfreq. = minfreq. ⋅ factor*^i^* with i *∈ {*0,1,…,29*}* and factor = ${\frac{\text{maxfreq}}{\text{minfreq}}}^{1/\left(\mathrm{channels}-1\right)}$]. Thus, for higher frequencies the spacing of the center frequencies becomes larger in analogy to the tonotopy of the human cochlea (Kandel et al., 2000; Fox, 2006). The width of the bandpass filters is defined as [centerfreq. factor^–0.5^, centerfreq. factor^0.5^].

### Model of the Dorsal Cochlear Nucleus

The dorsal cochlear nucleus (DCN) was modeled as 30 leaky integrate-and-fire (LIF) neurons (Burkitt, 2006), each of these neurons is connected to one frequency channel of the cochlea. Thus, no lateral inhibition was realized to focus on the core effects. The maximum spiking rate of the simulated LIF neurons is approximately 4 kHz (*t*_refrac_. = $\frac{11}{44100\,\frac{1}{\text{s}}}$≈$\frac{11}{4000}$S = 0.25 ms, *t*_refrac_.: refractory time), which is much more than the maximum spiking rate of a biological neuron (400 Hz) (Nizami, 2002). Thus, in the simulation 1 LIF neuron represent approximately 10 real neurons. The recruitment of several neurons to increase the frequency range in which phase coupling is possible is a core concept within the dorsal cochlear nucleus (Langner, 1988). The numerical integration of the LIF neurons was performed using the “Euler” method, as this method lead to the lowest computational complexity compared to “Heun” and “Runge Kutte”—being standard integration techniques (Fathoni and Wuryandari, 2015)—without causing significant inaccuracies.

### Model of Brain Stem and Cortex

The neural processing stages of the auditory pathway above the DCN including superior olive, lateral lemniscus, inferior colliculus, medial corpus geniculatum in the thalamus, and auditory cortex are modeled as a deep neural network (Kandel et al., 2000). For our main simulations with the custom-made data set we used a Deep Convolutional Neural Network (LeCun et al., 2015) (for architecture and exact parameters see Table 1). For the FSDD data set we used a slightly different architecture (Table 2). Furthermore, we also used Deep LSTM networks (Hochreiter and Schmidhuber, 1997) to double-check the validity and universality of the beneficial effects of intrinsic noise (Table 3).

### Data Sets

#### Custom-Made Data Set

Our custom-made data set created for the purpose of the present study was recorded from 12 different speakers (6 male, 6 female) in a range of 20–61 years. The data was recorded with a sampling rate of 44.1 kHz bit using Audacity. Each participant had to speak the 207 most common German words 10 times each. After recording, the data was labeled using forced alignment and cut into 1 s intervals. The data from 10 participants served as training data set, whereas the data from the two other speakers was used as test data set. All evaluations, i.e., simulated hearing loss and effect of intrinsic noise, were based on the modified test data.

#### Ethics Statement

All experiments were performed in accordance with relevant guidelines and regulations. Informed consent was obtained from all subjects. According to the Ethics Committee of the University Hospital Erlangen, no further ethics approval was required since non-invasive studies like this are exempted from formal ethics approvals.

#### Free Spoken Digit Data Set Data Set

The second used data set is an open data set consisting of spoken digits (0–9)–in analogy to the MNIST data set– in English. The data set is sampled with 8 kHz and consists of 2,000 recorded digits from four speakers (Jackson et al., 2018). Here the first five repetitions of for each speaker and each digit are used as test data, the respective remaining 45 repetitions serve as training data.

### Training of Deep Neural Networks With Undisturbed Test Data

As described above the complete auditory pathway beyond the DCN, including the superior olive, lateral lemniscus, inferior colliculus, medial geniculate corpus, and the auditory cortex, is modeled as a deep neural network which is trained on the classification of 207 different German words (custom- made data set), or 10 English words corresponding to the digits from 0 to 9 (FSDD data set; Jackson et al., 2018), respectively. In both cases the compressed, i.e., down sampled, DCN output matrices served as training and test data input.

In case of our custom-made data set, the network is exclusively trained on the data of 10 out of 12 speakers, while the remaining two speakers serve as test data. Furthermore, for network training we used only those compressed spike train matrices that correspond to the undisturbed system, i.e., without hearing loss and added noise. Due to the image-like features of the compressed spike pattern matrices [similar to frequently used Mel spectrograms (Meng et al., 2019) in speech recognition, the deep neural network mainly consisted of convolutional layers. The exact architecture and all parameters are provided in Table 1]. For training on our custom-made data set, the maximum test accuracy (0.37) significantly decreases after 20 epochs of training, and thus we applied the *early stopping procedure* (Caruana et al., 2001) to prevent the network from overfitting. The trained networks were used for all further analyses with different modifications of the test data set, i.e., different hearing losses and different intensities of intrinsic noise.

### Simulation of Hearing Loss

The hearing loss was simulated by a linear attenuation of the cochlear output at the affected frequency ranges. Thus, a hearing loss of *X* dB means that the outputs of the affected frequency channels are multiplied with the factor $10^{\frac{\text{X}}{20}}$. Additionally, for further experiments we added a real information loss by setting an additional threshold. A threshold of -*X* dB means that all values, where the absolute value is smaller than the threshold value $10^{\frac{-X}{20}}$ are set to 0 (see Figure 9A).

## Discussion

In this study, we demonstrated with a computational model of the auditory system that noise added to the DCN may improve speech recognition after hearing loss, by means of SR. The relative benefit of SR turned out to be largest for hearing losses between 20 and 30 dB.

Because SR works by partly restoring missing information in the input data, adding noise improves the classification accuracy of the neural network even after the training period is finished. This stands in contrast to machine learning techniques that achieve an increased robustness and generalization ability by purposefully using noisy training data from the beginning (Karpukhin et al., 2019), or by adding artificial noise during the training period (An, 1996; Zhao et al., 2019).

Furthermore, this is also the crucial difference between the SR model of auditory perception and alternative central gain models. Instead of restoring the average spontaneous neural activity after hearing loss, SR increases the information transmitted to the auditory system.

In our work, we first train the neural network for speech recognition, then simulate a hearing loss, and finally reduce this loss by adding noise. This approach is biologically plausible, as also the brain is trained on speech recognition during childhood (Dabrowska and Kubinski, 2004; Gervain, 2015), where hearing ability is usually optimal [Indeed, hearing impairment in childhood can lead to problems in language acquisition, which cannot be fully cured in adulthood (Pimperton and Kennedy, 2012)]. In the coarse of a lifetime, hearing ability becomes permanently (Gates and Mills, 2005; Huang and Tang, 2010) or temporarily worse (Willott and Lu, 1982), often due to high amplitude sound exposure.

We have proposed that hearing ability can be restored by a control cycle embedded in the brain stem, along the auditory pathway, which uses internal neural noise to exploit the effect of stochastic resonance (Krauss et al., 2016). Thus, it is supposed that the neural activity in damaged frequency channels is up-regulated by internally generated noise to restore hearing within this frequency range. Indeed, simulated transient hearing loss improves auditory detection thresholds (Krauss and Tziridis, 2021).

Overshooting of this noise up-regulation is proposed to be the origin of tinnitus (Krauss et al., 2016). Our model could provide an interesting explanation for overshooting internal noise: In our simulation of high frequency hearing loss, we found that the accuracy as a function of the added noise has not only a single maximum, as expected for a resonance curve, but features a second maximum at a higher noise level (Figure 8B). If the neural control cycle would be drawn to this secondary maximum, this might explain an overshooting of the neural noise and the corresponding emergence of tinnitus (Krauss et al., 2016, 2017; Schilling et al., 2021d). Another potential cause of tinnitus arises from the fact that phase locking, the encoding of a signal’s phase information in neural spike trains, is only possible for frequencies up to 4 kHz, the maximum spike rate of the DCN neurons (Figure 3A).

The stochastic resonance effect probably works only below this limit frequency, and thus it is not clear whether (or how) the neural control system compensates for the hearing loss in the frequency range above 4 kHz, as it has no real maximum to optimize for. Potentially, the tuning of the noise parameters in this frequency regime is done only by random trial. This model would fit to the observation that tinnitus mainly occurs in the high frequency range (Gollnast et al., 2017).

While we propose the DCN to be the place where auditory input from the cochlea is integrated with neural noise from the somatosensory system, we cannot rule out that SR rather occurs in the ventral cochlear nucleus (VCN) instead. Our LIF neurons correspond to narrow band neurons, which transform their cochlear input with minimal processing into spike trains. Neurons like e.g., bushy cells with such primary-like responses that show increased spontaneous firing rate after hearing loss are known to exist also in the VCN (Martel and Shore, 2020). In contrast, DCN neurons show strong non-linearities in sound processing through inhibitory shaping of their responses by inhibitory inter-neurons (Young and Davis, 2002; Oertel and Young, 2004). This circuitry might be the correlate of the noise-adjusting feedback-loop proposed in our model. For the sake of simplicity, we did not explicitly model this exact circuitry. Furthermore, the VCN is also innervated by trigeminal nerve fibers (Wu et al., 2015, 2016) which may be the source of the neural noise for SR. However, the DCN identification is not necessary for our model to work, and the identification of our model LIF neurons as VCN neurons would be possible as well.

We were able to show that neural noise could potentially help to increase speech comprehension in neural systems in a computational model of the auditory pathway. Even though, previous studies suggested a benefit of SR of only about 5*dB* (Zeng et al., 2000; Krauss et al., 2016; Gollnast et al., 2017), an accuracy improvement of up to a factor of 2 is possible. This model provides new insights how the auditory system optimizes speech comprehension on small time scales, and why this processing was evolutionary preserved even though, tinnitus results in strong psychiatric burden: comprehension of natural speech (Schilling et al., 2021c; Garibyan et al., 2022) is essential for humans. More general, recognition of communication sounds can be assumed to be essential for all social species, in particular mammals. This may explain why behavioral and neural correlates of tinnitus are also frequently observed in rodents.

Furthermore, we could give a mechanistic explanation of the development and characteristics of tinnitus perception. These finding could have a major impact on medical treatment of phantom perceptions, but on the other hand raises new research questions in the field of engineering.

However, it has to be stated that the SR model of tinnitus development is by no means complete. While our model provides a valid explanation for acute tinnitus perceived directly after noise trauma, and also explains why a tinnitus percept could be suppressed by acoustic noise of low intensity (Schilling et al., 2021a; Tziridis et al., 2022), it does not include long-term neural circuit-level effects (Jeschke et al., 2021) due to neural plasticity. Furthermore, our model is [like the central gain (Auerbach et al., 2014) and the lateral inhibition model (Gerken, 1996)] a pure bottom-up model, which means that cortical or thalamocortical top-down modulations are not regarded. Note that, we do not discuss further bottom-up models of tinnitus development in detail, as these models make no predictions on speech perception benefit of tinnitus after hearing loss (for an in-depth comparison of the different models, see Schilling et al., 2022). In contrast to bottom-up models, top- down models play a crucial role in understanding why brainstem hyperactivity passes the “gate to consciousness” (the thalamus) and results in suffering a psychic burden. Furthermore, attention effects also play a crucial role in stress related modulations of tinnitus loudness (Mazurek et al., 2015). Thus, some models describe the conscious tinnitus percept as a consequence of thalamocortical dysrhythmia. This dysrhythmia is induced by changed thalamo-cortical signal transmission, which is a result of reduced resp. increased sub-thalamic input to the medial geniculate body (Llińas et al., 1999; De Ridder et al., 2015; Gault et al., 2018). More recent approaches suggest that tinnitus is a prediction error and formalize their models within the Bayesian brain framework (Sedley et al., 2016; Hullfish et al., 2019; De Ridder and Vanneste, 2021). In summary, it is necessary to merge bottom-up and top-down models of tinnitus development to achieve a unified explanation of tinnitus development (Schilling et al., 2022). Our bottom-up model has not exclusively explanatory power but might also serve as source of inspiration for advanced machine learning approaches.

Furthermore, the effect of SR could be used to improve sensory systems (Krauss et al., 2017). Although noisy data is already used to make machine learning approaches more stable and less vulnerable to small distortions (e.g., Neelakantan et al., 2015; Gulcehre et al., 2016), the SR phenomenon can be used in a different way. Thus, feedback loops could be implemented in artificial intelligence systems, which are optimized on finding the ideal noise level to make a signal detectable. This approach goes well beyond already established techniques in artificial intelligence research.

Our study provides evidence that an interplay of deep learning and neuroscience helps on the one hand to raise understanding of the function of biological neural networks and cognition in general (e.g., Schilling et al., 2018, 2021b; Krauss et al., 2019a,c,d, 2021; Gerum et al., 2020; Krauss and Maier, 2020; Bönsel et al., 2021; Metzner and Krauss, 2022), an emerging science strand referred to as cognitive computational neuroscience (Kriegeskorte and Douglas, 2018). On the other hand, fundamental processing principles from nature—such as stochastic resonance—can be transferred to improve artificial neural systems, which is called neuroscience-inspired AI (Hassabis et al., 2017; Gerum et al., 2020; Gerum and Schilling, 2021; Yang et al., 2021; Maier et al., 2022).

## Data Availability Statement

The raw data supporting the conclusions of this article will be made available by the authors, without undue reservation.

## Author Contributions

AS, RG, and PK performed the simulations. AS, RG, CM, and PK analyzed the data. CM and AM provided the analysis tools. PK and AM supervised the study. All authors wrote the manuscript.

## Conflict of Interest

The authors declare that the research was conducted in the absence of any commercial or financial relationships that could be construed as a potential conflict of interest.

## Publisher’s Note

All claims expressed in this article are solely those of the authors and do not necessarily represent those of their affiliated organizations, or those of the publisher, the editors and the reviewers. Any product that may be evaluated in this article, or claim that may be made by its manufacturer, is not guaranteed or endorsed by the publisher.

## References

[B1] Abadi M. Barham P. Chen J. Chen Z. Davis A. Dean J. (2016). “Tensorflow: a system for large-scale machine learning,” in *Proceedings of the 12th {USENIX} Symposium on Operating Systems Design and Implementation ({OSDI} 16)*, (Berkeley, CA: USENIX), 265–283.

[B2] Ahlf S. Tziridis K. Korn S. Strohmeyer I. Schulze H. (2012). Predisposition for and prevention of subjective tinnitus development. *PLoS One* 7:e44519. 10.1371/journal.pone.0044519 23056180 PMC3462765

[B3] Aihara T. Kitajo K. Nozaki D. Yamamoto Y. (2008). Internal noise determines external stochastic resonance in visual perception. *Vis. Res.* 48 1569–1573. 10.1016/j.visres.2008.04.022 18514251

[B4] An G. (1996). The effects of adding noise during backpropagation training on a generalization per- formance. *Neural Comput.* 8 643–674.

[B5] Ansorge J. Wu C. Shore S. E. Krieger P. (2021). Audiotactile interactions in the mouse cochlear nucleus. *Sci. Rep.* 11:6887. 10.1038/s41598-021-86236-9 33767295 PMC7994829

[B6] Auerbach B. D. Rodrigues P. V. Salvi R. J. (2014). Central gain control in tinnitus and hyperacusis. *Front. Neurol.* 5:206. 10.3389/fneur.2014.00206 25386157 PMC4208401

[B7] Baizer J. S. Manohar S. Paolone N. A. Weinstock N. Salvi R. J. (2012). Understanding tinnitus: the dorsal cochlear nucleus, organization and plasticity. *Brain Res.* 1485 40–53. 10.1016/j.brainres.2012.03.044 22513100 PMC3402636

[B8] Benzi R. Sutera A. Vulpiani A. (1981). The mechanism of stochastic resonance. *J. Phys. A Math. Gen.* 14:L453.

[B9] Brozoski T. Bauer C. Caspary D. (2002). Elevated fusiform cell activity in the dorsal cochlear nucleus of chinchillas with psychophysical evidence of tinnitus. *J. Neurosci.* 22 2383–2390. 10.1523/JNEUROSCI.22-06-02383.2002 11896177 PMC6758251

[B10] Burkitt A. N. (2006). A review of the integrate-and-fire neuron model: I. homogeneous synaptic input. *Biol. Cybern.* 95 1–19. 10.1007/s00422-006-0068-6 16622699

[B11] Bönsel F. Krauss P. Metzner C. Yamakou M. E. (2021). Control of noise-induced coherent oscillations in three-neuron motifs. *Cogn. Neurodyn.* 636, 1–20. 10.1007/s11571-021-09770-2 PMC9279551 35847543

[B12] Carney L. H. (1993). A model for the responses of low-frequency auditory-nerve fibers in cat. *J. Acoust. Soc. Am.* 93 401–417. 10.1121/1.405620 8423257

[B13] Carney L. H. (2021). Speeding up machine hearing. *Nat. Mach. Intell.* 3 190–191.

[B14] Caruana R. Lawrence S. Giles C. L. (2001). “Overfitting in neural nets: backpropagation, conjugate gradient, and early stopping,” in *Proceedings of the Advances in Neural Information Processing Systems*, (Cambridge, MA: MIT Press), 402–408.

[B15] Chambers J. Elgueda D. Fritz J. B. Shamma S. A. Burkitt A. N. Grayden D. B. (2019). Computational neural modelling of auditory cortical receptive fields. *Front. Comput. Neurosci.* 13:28. 10.3389/fncom.2019.00028 31178710 PMC6543553

[B16] Chollet F. (2018). *Deep Learning mit Python und Keras: Das Praxis-Handbuch vom Entwickler der Keras-Bibliothek.* Wachtendonk: MITP Verlags-GmbH & Co. KG.

[B17] Ciorba A. Benatti A. Bianchini C. Aimoni C. Volpato S. Bovo R. (2011). High frequency hearing loss in the elderly: effect of age and noise exposure in an Italian group. *J. Laryngol. Otol.* 125 776–780. 10.1017/S0022215111001101 21729437

[B18] Collins J. J. Imhoff T. T. Grigg P. (1996). Noise-enhanced information transmission in rat sa1 cutaneous mechanoreceptors via aperiodic stochastic resonance. *J. Neurophysiol.* 76 642–645. 10.1152/jn.1996.76.1.642 8836253

[B19] Dabrowska E. Kubinski W. (2004). Language acquisition in the light of cognitive linguistics. Zmogus Kalbos Erdveje [Man in the Space of Language]. *Moksliniu Straipsniu Rinkinys* 3 253–267.

[B20] Dallos P. (1992). The active cochlea. *J. Neurosci.* 12 4575–4585. 1464757 10.1523/JNEUROSCI.12-12-04575.1992 PMC6575778

[B21] De Ridder D. Vanneste S. (2021). The bayesian brain in imbalance: medial, lateral and descending pathways in tinnitus and pain: a perspective. *Prog. Brain Res.* 262 309–334. 10.1016/bs.pbr.2020.07.012 33931186

[B22] De Ridder D. Vanneste S. Langguth B. Llinas R. (2015). Thalamocortical dysrhythmia: a theoretical update in tinnitus. *Front. Neurol.* 6:124. 10.3389/fneur.2015.00124 26106362 PMC4460809

[B23] Dehmel S. Pradhan S. Koehler S. Bledsoe S. Shore S. (2012). Noise overexposure alters long-term somatosensory-auditory processing in the dorsal cochlear nucleus—possible basis for tinnitus-related hyperactivity? *J. Neurosci.* 32 1660–1671. 10.1523/JNEUROSCI.4608-11.2012 22302808 PMC3567464

[B24] Douglass J. K. Wilkens L. Pantazelou E. Moss F. (1993). Noise enhancement of information transfer in crayfish mechanoreceptors by stochastic resonance. *Nature* 365 337–340. 10.1038/365337a0 8377824

[B25] Faisal A. A. Selen L. P. Wolpert D. M. (2008). Noise in the nervous system. *Nat. Rev. Neurosci.* 9 292–303. 18319728 10.1038/nrn2258 PMC2631351

[B26] Fathoni M. F. Wuryandari A. I. (2015). “Comparison between euler, heun, runge-kutta and adams-bashforth-moulton integration methods in the particle dynamic simulation,” in *Proceedings of the 2015 4th International Conference on Interactive Digital Media (ICIDM)* (Johor Bahru: IEEE), 1–7.

[B27] Fox S. I. (2006). *Human Physiology*, 9th Edn. New York, NY: McGraw-Hill press.

[B28] Gammaitoni L. Hänggi P. Jung P. Marchesoni F. (1998). Stochastic resonance. *Rev. Mod. Phys.* 70 223–287.

[B29] Gao Y. Manzoor N. Kaltenbach J. (2016). Evidence of activity-dependent plasticity in the dorsal cochlear nucleus, in vivo, induced by brief sound exposure. *Hear. Res.* 341 31–42. 10.1016/j.heares.2016.07.011 27490001 PMC5086438

[B30] Garibyan A. Schilling A. Boehm C. Zankl A. Krauss P. (2022). Neural correlates of linguistic collocations during continuous speech perception. *bioRxiv* [Preprint] 10.1101/2022.03.25.485771 PMC9822706 36619132

[B31] Gates G. A. Mills J. H. (2005). Presbycusis. *Lancet* 366 1111–1120. 16182900 10.1016/S0140-6736(05)67423-5

[B32] Gault R. Mcginnity T. M. Coleman S. (2018). A computational model of thalamocortical dysrhythmia in people with tinnitus. *IEEE Trans. Neural Syst. Rehabil. Eng.* 26 1845–1857. 10.1109/TNSRE.2018.2863740 30106678

[B33] Gerken G. M. (1996). Central tinnitus and lateral inhibition: an auditory brainstem model. *Hear. Res.* 97 75–83. 8844188

[B34] Gerum R. (2020). Pylustrator: code generation for reproducible figures for publication. *J. Open Source Softw.* 5:1989.

[B35] Gerum R. C. Erpenbeck A. Krauss P. Schilling A. (2020). Sparsity through evolutionary pruning prevents neuronal networks from overfitting. *Neural Netw.* 128 305–312. 10.1016/j.neunet.2020.05.007 32454374

[B36] Gerum R. C. Schilling A. (2021). Integration of leaky-integrate-and-fire neurons in standard machine learning architectures to generate hybrid networks: a surrogate gradient approach. *Neural Comput.* 33 2827–2852. 10.1162/neco_a_01424 34280298

[B37] Gervain J. (2015). Plasticity in early language acquisition: the effects of prenatal and early childhood experience. *Curr. Opin. Neurobiol.* 35 13–20. 10.1016/j.conb.2015.05.004 26093365

[B38] Gluckman B. J. Netoff T. I. Neel E. J. Ditto W. L. Spano M. L. Schiff S. J. (1996). Stochastic resonance in a neuronal network from mammalian brain. *Phys. Rev. Lett.* 77 4098–4101. 10.1103/PhysRevLett.77.4098 10062387

[B39] Gollnast D. Tziridis K. Krauss P. Schilling A. Hoppe U. Schulze H. (2017). Analysis of audiometric differences of patients with and without tinnitus in a large clinical database. *Front. Neurol.* 8:31. 10.3389/fneur.2017.00031 28232817 PMC5298966

[B40] Gulcehre C. Moczulski M. Denil M. Bengio Y. (2016). “Noisy activation functions,” in *Proceedings of the International Conference on Machine Learning*, (London: PMLR), 3059–3068.

[B41] Hackney C. M. Osen K. K. Kolston J. (1990). Anatomy of the cochlear nuclear complex of guinea pig. *Anat. Embryol.* 182 123–149. 10.1007/BF00174013 2244686

[B42] Hassabis D. Kumaran D. Summerfield C. Botvinick M. (2017). Neuroscience-inspired artificial intelligence. *Neuron* 95 245–258. 28728020 10.1016/j.neuron.2017.06.011

[B43] Heller A. J. (2003). Classification and epidemiology of tinnitus. *Otolaryngol. Clin. North Am.* 36 239–248. 10.1016/s0030-6665(02)00160-3 12856294

[B44] Hochreiter S. Schmidhuber J. (1997). Long short-term memory. *Neural Comput.* 9 1735–1780. 9377276 10.1162/neco.1997.9.8.1735

[B45] Houser D. S. Helweg D. A. Moore P. W. (2001). A bandpass filter-bank model of auditory sensitivity in the humpback whale. *Aquat. Mamm.* 27 82–91.

[B46] Huang J. Lu T. Sheffield B. Zeng F.-G. (2019). Electro-tactile stimulation enhances cochlear- implant melody recognition: effects of rhythm and musical training. *Ear Hear.* 41 106–113. 10.1097/AUD.0000000000000749 31884501

[B47] Huang J. Sheffield B. Lin P. Zeng F.-G. (2017). Electro-tactile stimulation enhances cochlear implant speech recognition in noise. *Sci. Rep.* 7:2196. 10.1038/s41598-017-02429-1 28526871 PMC5438362

[B48] Huang Q. Tang J. (2010). Age-related hearing loss or presbycusis. *Eur. Arch. Otorhinolaryngol.* 267 1179–1191. 20464410 10.1007/s00405-010-1270-7

[B49] Hullfish J. Sedley W. Vanneste S. (2019). Prediction and perception: insights for (and from) tinnitus. *Neurosci. Biobehav. Rev.* 102 1–12. 10.1016/j.neubiorev.2019.04.008 30998951

[B50] Hunter J. D. (2007). Matplotlib: a 2d graphics environment. *Comput. Sci. Eng.* 9 90–95.

[B51] Jackson Z. Souza C. Flaks J. Pan Y. Nicolas H. Thite A. (2018). Jakobovski/free-spoken- digit-dataset: v1.0.8. 10.5281/zenodo.1342401

[B52] James R. Garside J. Plana L. A. Rowley A. Furber S. B. (2018). Parallel distribution of an inner hair cell and auditory nerve model for real-time application. *IEEE Trans. Biomed. Circuits Syst.* 12 1018–1026. 10.1109/TBCAS.2018.2847562 30010597

[B53] Jeschke M. Happel M. F. Tziridis K. Krauss P. Schilling A. Schulze H. (2021). Acute and long-term circuit-level effects in the auditory cortex after sound trauma. *Front. Neurosci.* 14:598406. 10.3389/fnins.2020.598406 33469416 PMC7813782

[B54] Jones E. Oliphant T. Peterson P. (2001). *Scipy: Open Source Scientific Tools for Python.* Available Online at: http://www.scipy.org (accessed May 27, 2022).

[B55] Kaltenbach J. A. Afman C. E. (2000). Hyperactivity in the dorsal cochlear nucleus after intense sound exposure and its resemblance to tone-evoked activity: a physiological model for tinnitus. *Hear. Res.* 140 165–172. 10.1016/s0378-5955(99)00197-5 10675644

[B56] Kaltenbach J. A. Godfrey D. A. Neumann J. B. McCaslin D. L. Afman C. E. Zhang J. (1998). Changes in spontaneous neural activity in the dorsal cochlear nucleus following exposure to intense sound: relation to threshold shift. *Hear. Res.* 124 78–84. 10.1016/s0378-5955(98)00119-1 9822904

[B57] Kaltenbach J. A. Rachel J. D. Mathog T. A. Zhang J. Falzarano P. R. Lewandowski M. (2002). Cisplatin-induced hyperactivity in the dorsal cochlear nucleus and its relation to outer hair cell loss: relevance to tinnitus. *J. Neurophysiol.* 88 699–714. 10.1152/jn.2002.88.2.699 12163523

[B58] Kaltenbach J. A. Zacharek M. A. Zhang J. Frederick S. (2004). Activity in the dorsal cochlear nucleus of hamsters previously tested for tinnitus following intense tone exposure. *Neurosci. Lett.* 355 121–125. 10.1016/j.neulet.2003.10.038 14729250

[B59] Kandel E. R. Schwartz J. H. Jessell T. M. Jessell M. B. T. Siegelbaum S. Hudspeth A. (2000). *Principles of Neural Science*, Volume 4. New York, NY: McGraw-hill.

[B60] Karpukhin V. Levy O. Eisenstein J. Ghazvininejad M. (2019). Training on synthetic noise improves robustness to natural noise in machine translation. *arXiv* [Preprint] 10.48550/arXiv.1902.01509

[B61] Koehler S. D. Shore S. E. (2013). Stimulus timing-dependent plasticity in dorsal cochlear nucleus is altered in tinnitus. *J. Neurosci.* 33 19647–19656. 10.1523/JNEUROSCI.2788-13.2013 24336728 PMC3858633

[B62] Koops E. A. Eggermont J. J. (2021). The thalamus and tinnitus: bridging the gap between animal data and findings in humans. *Hear. Res.* 407:108280. 10.1016/j.heares.2021.108280 34175683

[B63] Kosko B. Mitaim S. (2003). Stochastic resonance in noisy threshold neurons. *Neural Netw.* 16 755–761. 10.1016/S0893-6080(03)00128-X 12850031

[B64] Krauss P. Maier A. (2020). Will we ever have conscious machines? *Front. Comput. Neurosci.* 14:556544. 10.3389/fncom.2020.556544 33414712 PMC7782472

[B65] Krauss P. Metzner C. Joshi N. Schulze H. Traxdorf M. Maier A. (2021). Analysis and visualization of sleep stages based on deep neural networks. *Neurobiol. Sleep Circadian Rhythms* 10:100064. 10.1016/j.nbscr.2021.100064 33763623 PMC7973384

[B66] Krauss P. Metzner C. Schilling A. Schütz C. Tziridis K. Fabry B. (2017). Adaptive stochastic resonance for unknown and variable input signals. *Sci. Rep.* 7:2450. 10.1038/s41598-017-02644-w 28550314 PMC5446399

[B67] Krauss P. Prebeck K. Schilling A. Metzner C. (2019a). Recurrence resonance” in three-neuron motifs. *Front. Comput. Neurosci.* 13:64. 10.3389/fncom.2019.00064 31572152 PMC6749061

[B68] Krauss P. Schilling A. Tziridis K. Schulze H. (2019b). Models of tinnitus development: from cochlea to cortex. *HNO* 67 172–177. 10.1007/s00106-019-0612-z 30707242

[B69] Krauss P. Schuster M. Dietrich V. Schilling A. Schulze H. Metzner C. (2019c). Weight statistics controls dynamics in recurrent neural networks. *PLoS One* 14:e0214541. 10.1371/journal.pone.0214541 30964879 PMC6456246

[B70] Krauss P. Zankl A. Schilling A. Schulze H. Metzner C. (2019d). Analysis of structure and dynamics in three-neuron motifs. *Front. Comput. Neurosci.* 13:5. 10.3389/fncom.2019.00005 30792635 PMC6374328

[B71] Krauss P. Schilling A. (2020). Towards a cognitive computational neuroscience of auditory phantom perceptions. *arXiv* [Preprint] 10.48550/arXiv.2010.01914

[B72] Krauss P. Tziridis K. (2021). Simulated transient hearing loss improves auditory sensitivity. *Sci. Rep.* 11:14791. 10.1038/s41598-021-94429-5 34285327 PMC8292442

[B73] Krauss P. Tziridis K. Metzner C. Schilling A. Hoppe U. Schulze H. (2016). Stochastic resonance controlled upregulation of internal noise after hearing loss as a putative cause of tinnitus- related neuronal hyperactivity. *Front. Neurosci.* 10:597. 10.3389/fnins.2016.00597 28082861 PMC5187388

[B74] Krauss P. Tziridis K. Schilling A. Schulze H. (2018). Cross-modal stochastic resonance as a universal principle to enhance sensory processing. *Front. Neurosci.* 12:578. 10.3389/fnins.2018.00578 30186104 PMC6110899

[B75] Kriegeskorte N. Douglas P. K. (2018). Cognitive computational neuroscience. *Nat. Neurosci.* 21 1148–1160. 30127428 10.1038/s41593-018-0210-5 PMC6706072

[B76] König O. Schaette R. Kempter R. Gross M. (2006). Course of hearing loss and occurrence of tinnitus. *Hear. Res.* 221 59–64. 10.1016/j.heares.2006.07.007 16962270

[B77] Langner G. (1988). “Physiological properties of units in the cochlear nucleus are adequate for a model of periodicity analysis in the auditory midbrain,” in *Auditory Pathway*, eds Syka J. Masterton R. B. (Boston, MA: Springer), 207–212.

[B78] LeCun Y. Bengio Y. Hinton G. (2015). Deep learning. *Nature* 521 436–444. 26017442 10.1038/nature14539

[B79] LeCun Y. Bottou L. Bengio Y. Haffner P. (1998). Gradient-based learning applied to document recognition. *Proc. IEEE* 86 2278–2324.

[B80] Levin J. E. Miller J. P. (1996). Broadband neural encoding in the cricket cereal sensory system enhanced by stochastic resonance. *Nature* 380 165–168. 10.1038/380165a0 8600392

[B81] Levine R. A. (1999). Somatic (craniocervical) tinnitus and the dorsal cochlear nucleus hypothesis. *Am. J. Otolaryngol.* 20 351–362. 10.1016/s0196-0709(99)90074-1 10609479

[B82] Liberman L. D. Liberman M. C. (2015). Dynamics of cochlear synaptopathy after acoustic overexposure. *J. Assoc. Res. Otolaryngol.* 16 205–219. 25676132 10.1007/s10162-015-0510-3 PMC4368657

[B83] Liberman M. C. Epstein M. J. Cleveland S. S. Wang H. Maison S. F. (2016). Toward a differential diagnosis of hidden hearing loss in humans. *PLoS One* 11:e0162726. 10.1371/journal.pone.0162726 27618300 PMC5019483

[B84] Licklider J. C. R. (1951). A duplex theory of pitch perception. *J. Acoust. Soc. Am.* 23:147. 10.1007/BF02156143 14831572

[B85] Llińas R. R. Ribary U. Jeanmonod D. Kronberg E. Mitra P. P. (1999). Thalamocortical dysrhythmia: a neurological and neuropsychiatric syndrome characterized by magnetoencephalography. *Proc. Natl. Acad. Sci. U.S.A.* 96 15222–15227. 10.1073/pnas.96.26.15222 10611366 PMC24801

[B86] Lorenzi C. Gilbert G. Carn H. Garnier S. Moore B. C. (2006). Speech perception problems of the hearing impaired reflect inability to use temporal fine structure. *Proc. Natl. Acad. Sci. U.S.A.* 103 18866–18869. 10.1073/pnas.0607364103 17116863 PMC1693753

[B87] Maier A. Köstler H. Heisig M. Krauss P. Yang S. H. (2022). Known operator learning and hybrid machine learning in medical imaging—a review of the past, the present, and the future. *Prog. Biomed. Eng.* 4:022002.

[B88] Marr D. Poggio T. (1979). A computational theory of human stereo vision. *Proc. R. Soc. Lond. B Biol. Sci.* 204 301–328. 10.1098/rspb.1979.0029 37518

[B89] Martel D. T. Shore S. E. (2020). Ventral cochlear nucleus bushy cells encode hyperacusis in guinea pigs. *Sci. Rep.* 10:20594. 10.1038/s41598-020-77754-z 33244141 PMC7693270

[B90] Mazurek B. Szczepek A. Hebert S. (2015). Stress and tinnitus. *HNO* 63 258–265. 25862619 10.1007/s00106-014-2973-7

[B91] McDonnell M. D. Abbott D. (2009). What is stochastic resonance? definitions, misconceptions, debates, and its relevance to biology. *PLoS Comput. Biol.* 5:e1000348. 10.1371/journal.pcbi.1000348 19562010 PMC2660436

[B92] Meng H. Yan T. Yuan F. Wei H. (2019). Speech emotion recognition from 3d log-mel spectrograms with deep learning network. *IEEE Access* 7 125868–125881.

[B93] Metzner C. Krauss P. (2022). Dynamics and information import in recurrent neural networks. *Front. Comput. Neurosci.* 16:876315. 10.3389/fncom.2022.876315 35573264 PMC9091337

[B94] Mino H. (2014). The effects of spontaneous random activity on information transmission in an auditory brain stem neuron model. *Entropy* 16 6654–6666.

[B95] Mitaim S. Kosko B. (1998). Adaptive stochastic resonance. *Proc. IEEE* 86 2152–2183.

[B96] Mitaim S. Kosko B. (2004). Adaptive stochastic resonance in noisy neurons based on mutual information. *IEEE Trans. Neural Netw.* 15 1526–1540. 10.1109/TNN.2004.826218 15565779

[B97] Moore B. C. Glasberg B. R. (1983). Suggested formulae for calculating auditory-filter bandwidths and excitation patterns. *J. Acoust. Soc. Am.* 74 750–753. 10.1121/1.389861 6630731

[B98] Moss F. Ward L. M. Sannita W. G. (2004). Stochastic resonance and sensory information processing: a tutorial and review of application. *Clin. Neurophysiol.* 115 267–281. 10.1016/j.clinph.2003.09.014 14744566

[B99] Nadol J. B. Jr. (1988). Comparative anatomy of the cochlea and auditory nerve in mammals. *Hear. Res.* 34 253–266. 10.1016/0378-5955(88)90006-8 3049492

[B100] Neelakantan A. Vilnis L. Le Q. V. Sutskever I. Kaiser L. Kurach K. (2015). Adding gradient noise improves learning for very deep networks. *arXiv* [Preprint] 10.48550/arXiv.1511.06807

[B101] Nelken I. Young E. D. (1994). Two separate inhibitory mechanisms shape the responses of dorsal cochlear nucleus type IV units to narrowband and wideband stimuli. *J. Neurophysiol.* 71 2446–2462. 10.1152/jn.1994.71.6.2446 7931527

[B102] Nelken I. Young E. D. (1996). Why do cats need a dorsal cochlear nucleus? *J. Basic Clin. Physiol. Pharmacol.* 7 199–220. 10.1515/jbcpp.1996.7.3.199 8910137

[B103] Nelson J. J. Chen K. (2004). The relationship of tinnitus, hyperacusis, and hearing loss. *Ear Nose Throat J.* 83 472–476. 15372918

[B104] Niven E. C. Scott S. K. (2021). Careful whispers: when sounds feel like a touch. *Trends Cogn. Sci.* 25 645–647. 10.1016/j.tics.2021.05.006 34144894

[B105] Nizami L. (2002). Estimating auditory neuronal dynamic range using a fitted function. *Hear. Res.* 167 13–27. 10.1016/s0378-5955(02)00293-9 12117526

[B106] Nozaki D. Mar D. J. Grigg P. Collins J. J. (1999). Effects of colored noise on stochastic resonance in sensory neurons. *Phys. Rev. Lett.* 82:2402.

[B107] Oertel D. Young E. D. (2004). What’s a cerebellar circuit doing in the auditory system? *Trends Neurosci.* 27 104–110. 10.1016/j.tins.2003.12.001 15102490

[B108] Osen K. K. Syka J. Masterton R. B. (1988). “Anatomy of the mammalian cochlear nuclei; a review,” in *Auditory Pathway*, eds Syka J. Masterton R. B. (Boston, MA: Springer), 65–75.

[B109] Parra L. C. Pearlmutter B. A. (2007). Illusory percepts from auditory adaptation. *J. Acoust. Soc. Am.* 121 1632–1641. 10.1121/1.2431346 17407900

[B110] Pikovsky A. S. Kurths J. (1997). Coherence resonance in a noise-driven excitable system. *Phys. Rev. Lett.* 78:775.

[B111] Pimperton H. Kennedy C. R. (2012). The impact of early identification of permanent child- hood hearing impairment on speech and language outcomes. *Arch. Dis. Child.* 97 648–653. 10.1136/archdischild-2011-301501 22550319

[B112] Pinchoff R. J. Burkard R. F. Salvi R. J. Coad M. L. Lockwood A. H. (1998). Modulation of tinnitus by voluntary jaw movements. *Am. J. Otol.* 19 785–789. 9831155

[B113] Russell I. Nilsen K. (1997). The location of the cochlear amplifier: spatial representation of a single tone on the guinea pig basilar membrane. *Proc. Natl. Acad. Sci. U.S.A.* 94 2660–2664. 10.1073/pnas.94.6.2660 9122252 PMC20145

[B114] Ryugo D. K. Haenggeli C.-A. Doucet J. R. (2003). Multimodal inputs to the granule cell domain of the cochlear nucleus. *Exp. Brain Res.* 153 477–485. 10.1007/s00221-003-1605-3 13680048

[B115] Sayles M. Winter I. M. (2010). Equivalent-rectangular bandwidth of single units in the anaesthetized guinea-pig ventral cochlear nucleus. *Hear. Res.* 262 26–33. 10.1016/j.heares.2010.01.015 20123119

[B116] Schaette R. McAlpine D. (2011). Tinnitus with a normal audiogram: physiological evidence for hidden hearing loss and computational model. *J. Neurosci.* 31 13452–13457. 10.1523/JNEUROSCI.2156-11.2011 21940438 PMC6623281

[B117] Schilling A. Tziridis K. Schulze H. Krauss P. (2021d). The stochastic resonance model of auditory perception: a unified explanation of tinnitus development, zwicker tone illusion, and residual inhibition. *Prog. Brain Res.* 262 139–157. 10.1016/bs.pbr.2021.01.025 33931176

[B118] Schilling A. Tomasello R. Henningsen-Schomers M. R. Zankl A. Surendra K. Haller M. (2021c). Analysis of continuous neuronal activity evoked by natural speech with computational corpus linguistics methods. *Lang. Cogn. Neurosci.* 36 167–186.

[B119] Schilling A. Krauss P. Hannemann R. Schulze H. Tziridis K. (2021a). Reduktion der tinnituslautstärke: pilotstudie zur abschwächung von tonalem tinnitus mit schwellennahem, individu- ell spektral optimiertem rauschen. *HNO* 69:891. 10.1007/s00106-020-00963-5 PMC8545742 33185745

[B120] Schilling A. Maier A. Gerum R. Metzner C. Krauss P. (2021b). Quantifying the separability of data classes in neural networks. *Neural Netw.* 139 278–293. 10.1016/j.neunet.2021.03.035 33862387

[B121] Schilling A. Metzner C. Rietsch J. Gerum R. Schulze H. Krauss P. (2018). How deep is deep enough?–quantifying class separability in the hidden layers of deep neural networks. *arXiv* [Preprint] 10.48550/arXiv.1811.01753

[B122] Schilling A. Sedley W. Gerum R. Metzner C. Tziridis K. Maier A. (2022). Predictive coding and stochastic resonance: towards a unified theory of auditory (phantom) perception. *arXiv* [Preprint] 10.48550/arXiv.2204.03354 PMC10690027 37503725

[B123] Sedley W. Friston K. J. Gander P. E. Kumar S. Griffiths T. D. (2016). An integrative tinnitus model based on sensory precision. *Trends Neurosci.* 39 799–812. 10.1016/j.tins.2016.10.004 27871729 PMC5152595

[B124] Shannon C. E. (1948). A mathematical theory of communication. *Bell Syst. Tech. J.* 27 379–423.

[B125] Shore S. (2011). Plasticity of somatosensory inputs to the cochlear nucleus–implications for tinnitus. *Hear. Res.* 281 38–46. 10.1016/j.heares.2011.05.001 21620940 PMC3174344

[B126] Shore S. Koehler S. Oldakowski M. Hughes L. Syed S. (2008). Dorsal cochlear nucleus responses to somatosensory stimulation are enhanced after noise-induced hearing loss. *Eur. J. Neurosci.* 27 155–168. 10.1111/j.1460-9568.2007.05983.x 18184319 PMC2614620

[B127] Shore S. E. Roberts L. E. Langguth B. (2016). Maladaptive plasticity in tinnitus—triggers, mechanisms and treatment. *Nat. Rev. Neurol.* 12 150–160. 10.1038/nrneurol.2016.12 26868680 PMC4895692

[B128] Shore S. E. Zhou J. (2006). Somatosensory influence on the cochlear nucleus and beyond. *Hear. Res.* 216 90–99. 10.1016/j.heares.2006.01.006 16513306

[B129] Sumner C. J. Lopez-Poveda E. A. O’Mard L. P. Meddis R. (2002). A revised model of the inner-hair cell and auditory-nerve complex. *J. Acoust. Soc. Am.* 111 2178–2188. 10.1121/1.1453451 12051437

[B130] Tan Q. Carney L. H. (2003). A phenomenological model for the responses of auditory-nerve fibers. II. nonlinear tuning with a frequency glide. *J. Acoust. Soc. Am.* 114 2007–2020. 10.1121/1.1608963 14587601

[B131] Tang Z.-Q. Trussell L. O. (2015). Serotonergic regulation of excitability of principal cells of the dorsal cochlear nucleus. *J. Neurosci.* 35 4540–4551. 10.1523/JNEUROSCI.4825-14.2015 25788672 PMC4363383

[B132] Tang Z.-Q. Trussell L. O. (2017). Serotonergic modulation of sensory representation in a central multisensory circuit is pathway specific. *Cell Rep.* 20 1844–1854. 10.1016/j.celrep.2017.07.079 28834748 PMC5600294

[B133] Tziridis K. Ahlf S. Jeschke M. Happel M. F. Ohl F. W. Schulze H. (2015). Noise trauma induced neural plasticity throughout the auditory system of mongolian gerbils: differences between tinnitus developing and non-developing animals. *Front. Neurol.* 6:22. 10.3389/fneur.2015.00022 25713557 PMC4322711

[B134] Tziridis K. Brunner S. Schilling A. Krauss P. Schulze H. (2022). Spectrally matched near- threshold noise for subjective tinnitus loudness attenuation based on stochastic resonance. *Front. Neurosci.* 16:831581. 10.3389/fnins.2022.831581 35431789 PMC9005796

[B135] Tziridis K. Forster J. Buchheidt-Dörfler I. Krauss P. Schilling A. Wendler O. (2021). Tinnitus development is associated with synaptopathy of inner hair cells in mongolian gerbils. *Eur. J. Neurosci.* 54 4768–4780. 10.1111/ejn.15334 34061412

[B136] Usher M. Feingold M. (2000). Stochastic resonance in the speed of memory retrieval. *Biol. Cybernet.* 83 L011–L016. 10.1007/PL00007974 11130587

[B137] Verhulst S. Altoe A. Vasilkov V. (2018). Computational modeling of the human auditory periphery: auditory-nerve responses, evoked potentials and hearing loss. *Hear. Res.* 360 55–75. 10.1016/j.heares.2017.12.018 29472062

[B138] Walt S. V. D. Colbert S. C. Varoquaux G. (2011). The numPy array: a structure for efficient numerical computation. *Comput. Sci. Eng.* 13 22–30.

[B139] Wang J. Powers N. Hofstetter P. Trautwein P. Ding D. Salvi R. (1997). Effects of selective inner hair cell loss on auditory nerve fiber threshold, tuning and spontaneous and driven discharge rate. *Hear. Res.* 107 67–82. 10.1016/s0378-5955(97)00020-8 9165348

[B140] Ward L. M. Neiman A. Moss F. (2002). Stochastic resonance in psychophysics and in animal behavior. *Biol. Cybernet.* 87 91–101. 12181585 10.1007/s00422-002-0328-z

[B141] Wenning G. Obermayer K. (2003). Activity driven adaptive stochastic resonance. *Phys. Rev. Lett.* 90:120602. 10.1103/PhysRevLett.90.120602 12688861

[B142] Wiegrebe L. Kössl M. Schmidt S. (1996). Auditory enhancement at the absolute threshold of hearing and its relationship to the zwicker tone. *Hear. Res.* 100 171–180. 10.1016/0378-5955(96)00111-6 8922992

[B143] Wiesenfeld K. Moss F. (1995). Stochastic resonance and the benefits of noise: from ice ages to crayfish and squids. *Nature* 373 33–36. 10.1038/373033a0 7800036

[B144] Willott J. F. Lu S.-M. (1982). Noise-induced hearing loss can alter neural coding and increase excitability in the central nervous system. *Science* 216 1331–1334. 10.1126/science.7079767 7079767

[B145] Wu C. Stefanescu R. A. Martel D. T. Shore S. E. (2015). Listening to another sense: somatosensory integration in the auditory system. *Cell Tissue Res.* 361 233–250. 10.1007/s00441-014-2074-7 25526698 PMC4475675

[B146] Wu C. Stefanescu R. A. Martel D. T. Shore S. E. (2016). Tinnitus: maladaptive auditory– somatosensory plasticity. *Hear. Res.* 334 20–29. 10.1016/j.heares.2015.06.005 26074307 PMC4676957

[B147] Yang Z. Schilling A. Maier A. Krauss P. (2021). “Neural networks with fixed binary random projections improve accuracy in classifying noisy data,” in *Bildverarbeitung für die Medizin 2021*, eds Palm C. Deserno T. M. Handels H. Maier A. Maier-Hein K. Tolxdorff T. (Berlin: Springer), 211–216.

[B148] Young E. D. Davis K. A. (2002). “Circuitry and function of the dorsal cochlear nucleus,” in *Integrative Functions in the Mammalian Auditory Pathway*, eds Oertel D. Fay R. R. Popper A. N. (New York, NY: Springer), 160–206.

[B149] Young E. D. Nelken I. Conley R. A. (1995). Somatosensory effects on neurons in dorsal cochlear nucleus. *J. Neurophysiol.* 73 743–765. 7760132 10.1152/jn.1995.73.2.743

[B150] Zacharek M. A. Kaltenbach J. A. Mathog T. A. Zhang J. (2002). Effects of cochlear ablation on noise induced hyperactivity in the hamster dorsal cochlear nucleus: implications for the origin of noise induced tinnitus. *Hear. Res.* 172 137–144. 10.1016/s0378-5955(02)00575-0 12361876

[B151] Zeng C. Yang Z. Shreve L. Bledsoe S. Shore S. (2012). Somatosensory projections to cochlear nucleus are upregulated after unilateral deafness. *J. Neurosci.* 32 15791–15801. 10.1523/JNEUROSCI.2598-12.2012 23136418 PMC3501653

[B152] Zeng F.-G. (2013). An active loudness model suggesting tinnitus as increased central noise and hyperacusis as increased nonlinear gain. *Hear. Res.* 295 172–179. 10.1016/j.heares.2012.05.009 22641191 PMC3593089

[B153] Zeng F.-G. (2020). Tinnitus and hyperacusis: central noise, gain and variance. *Curr. Opin. Physiol.* 18 123–129. 10.1016/j.cophys.2020.10.009 33299958 PMC7720792

[B154] Zeng F.-G. Djalilian H. (2010). Hearing impairment. *Oxford Handb. Audit. Sci.* 3 325–347.

[B155] Zeng F.-G. Fu Q.-J. Morse R. (2000). Human hearing enhanced by noise. *Brain Res.* 869 251–255. 10.1016/s0006-8993(00)02475-6 10865084

[B156] Zeng F.-G. Liu S. (2006). Speech perception in individuals with auditory neuropathy. *J. Speech Lang. Hear. Res.* 49 367–380. 16671850 10.1044/1092-4388(2006/029)

[B157] Zhao C. Siguad O. Stulp F. Hospedales T. M. (2019). Investigating generalisation in continuous deep reinforcement learning. *arXiv* [Preprint] 10.48550/arXiv.1902.07015

[B158] Zwicker E. (1964). “Negative afterimage” in hearing. *J. Acoust. Soc. Am.* 36 2413–2415. 10.1121/1.1913052 5045252

